# Prevalence of Traumatic Dental Injuries to the Permanent Anterior Teeth among 9- to 14-year-old Schoolchildren of Navi Mumbai (Kharghar-Belapur Region), India

**DOI:** 10.5005/jp-journals-10005-1430

**Published:** 2017-06-01

**Authors:** Rahul Hegde, Geet Agrawal

**Affiliations:** 1 Professor and Head, Department of Pedodontics and Preventive Dentistry, Bharati Vidyapeeth Deemed University Dental College & Hospital, Navi Mumbai, Maharashtra, India; 2 Assistant Professor, Department of Pedodontics and Preventive Dentistry, Bharati Vidyapeeth Deemed University Dental College & Hospital, Navi Mumbai, Maharashtra, India

**Keywords:** Anterior teeth trauma, Dental trauma, Permanent anterior teeth, Risk factors, Traumatic dental injury.

## Abstract

**Aims and objectives:**

To measure the prevalence of anterior teeth trauma in 9- to 14-year-old schoolchildren and their association with predisposing factors, such as lip competence, molar relationship, overjet, and variables, such as age, gender, and cause of trauma.

**Materials and methods:**

Epidemiological cross-sectional study was carried out among 3,012 schoolchildren aged 9 to 14 years in Navi Mumbai (Kharghar-Belapur region). The sample size was derived using the stratified random sampling method; we picked six schools from the region. These schools had 3,000 students in the acceptable age group of the study which constituted our final sample size. All children were examined for traumatic dental injuries, and the children with positive findings were further examined for lip competence, Angle’s molar relationship, and overjet. The results were statistically analyzed using cross-tabulation and Chi-square test.

**Results:**

The prevalence of dental injuries was 7.3%, and the ratio of male to female children was found to be 1.6:1. The maximum number of traumatic dental injuries was found with class I molar relationship and overjet less than 3.5 mm in children with competent lips. Maximum number of injuries occurred in the age group of 13-14 years. The most predominant type of injury was the enamel fracture and the most common cause determined was due to fall.

**Conclusion:**

The present study is a collection of data on traumatic injuries to anterior teeth, which is seen very commonly in day-to-day practice. The frequency and cause of traumatic injuries to anterior teeth is important for identification of risk groups, treatment needs, and cost involved in order for establishing effective preventive measures.

**How to cite this article:**

Hegde R, Agrawal G. Prevalence of Traumatic Dental Injuries to the Permanent Anterior Teeth among 9- to 14-year-old Schoolchildren of Navi Mumbai (Kharghar-Belapur Region), India. Int J Clin Pediatr Dent 2017;10(2):177-182.

## INTRODUCTION

An individual’s quality of life is strongly influenced by their health condition. In the field of dental health, physical constraints may directly influence aspects of feeding, speech, social interaction and self-esteem, and psycho-logy.^1^ It is accepted that the position and appearance of the anterior teeth have psychological and social impact on children.^2^ Therefore, it is not surprising that dental problems are linked to social and psychological and emotional well-being. The contribution of dentofacial characteristics to dimensions of personality, self-esteem, and body image is well documented.^3^ Most of the literature found on dental injury pertains to the classification, incidence, prevalence, and treatment of fractured teeth. A major finding here is that the five leading causes of dentofacial injuries, regardless of severity, were falls, being struck by an object, bicycle accidents, assaults, and motor vehicle accidents.^4^ Trauma along with fracture of a permanent front tooth is a disturbing experience for the young patient and is a problem whose management requires experience, judgment, and skill perhaps incomparable to any other segment of the dental practice. The dentist whose counsel is sought after a trauma should treat the patient with all possible means or immediately refer the patient to a specialist. The oral and emotional health of the young patient is involved and the child’s appearance, marred by an unsightly oral injury, must be restored to normal as soon as possible to relieve the consciousness of being different from other children.^5^ Providing education to individuals who supervise and look after the children and would be in close proximity to the accident site of traumatic dental injury (TDI) has been widely advocated.^6^ These educational programs should explain the importance of prompt treatment for dental trauma, including ways of preventing these traumas and procedures for apt emergency management to be instituted. Such educational programs for the general public in a region should be preceded by an analysis of background information on the incidence of orodental injuries in that community.^7^

This served as the motivation for our present study to evaluate the prevalence of the traumatic injuries to the anterior teeth in schoolgoing children of Kharghar and Belapur region, Navi Mumbai, India, and to correlate the prevalence of injury to the cause of trauma with the age of the child, the incisal overjet, molar relation, and lip competence of the child.

## MATERIALS AND METHODS

The study consists of 3,012 primary and high schoolgoing children of both genders aged between 9 and 14 years selected by using stratified random sampling method. The sample size was derived from the population of 9- to 14-year-old schoolgoing children in the given area. There are currently 40,000 children studying in 22 schools in the Kharghar-Belapur region. Using the stratified random sampling method, we picked six schools from the region. These schools had 3,000 students in the acceptable age group of the study, which constituted our final sample size. Children with permanent anterior teeth lost due to caries or causes other than trauma or with partial or complete anodontia involving permanent anterior teeth were excluded. Informed consent was taken from the concerned school authorities and from all individual participants included in the study before the commencement of the survey. All the information regarding gender, age, number of injured teeth, type of the teeth, type of injury (treated and untreated), cause of injury, lip competence, the molar relationship, and overjet was carefully recorded. Children were divided in three age groups of 9 to 10, 11 to 12, and 13 to 14 years to find the age-wise prevalence of trauma. The examination was done using Andreasen’s epidemiological classification of TDIs including codes of the World Health Organization international classification of diseases to dentistry and stomatology (1997) by only one examiner. For the measurement of overjet, calibrations on the Community Periodontal Index of Treatment Needs probe were used, i.e., 3.5, 5.5, 8.5, and 11.5 mm. The children with TDIs were also observed for lip competence, and the molar relationships were recorded following Angle’s classification. The Federation Dentaire Internationale system of tooth numbering is followed in the study. The children were verbally asked to state the cause of the injury, which was documented under the categories of fall, collision, road accident, violence, cannot recollect, and miscellaneous. Miscellaneous category included biting on hard objects, such as pencil, pen, etc., and improper use of teeth, such as opening of bobby pins and soda pop bottles. The analysis of the data was done by using descriptive statistics (frequency distribution and cross-tabulation). Statistical significance of the association between the occurrence of the dental injuries and gender, age, cause, and overjet was tested using the chi-square test.

**Table 1:** **Table 1:** Descriptive statistics showing the cause of TDIs in males and females

		*Male*				*Female*				*Total*				Chi-square test*			
*Cause of injury*		*No.*		*%*		*No.*		*%%*		*No.*		*%%*		χ^2^		*p-value*	
Fall		83		61.10		53		63.20		136		61.8		4.197		0.040	
Collision		34		25		11		13		45		20.5		7.053		0.008	
Accident		6		4.40		5		6		11		5		0.015		0.902 (NS)	
Violence		6		4.40		1		1.10		7		3.2		1.237		0.266	
Cannot recollect		4		2.90		5		6		9		4.1		0.018		0.891	
Miscellaneous		3		2.30		9		10.70		12		5.4		1.157		0.282	
Total		136		100		84		100		220		100		7.776		0.005	

NS: Not significant; *Within gender comparison

**Table 2:** **Table 2:** Descriptive statistics showing the prevalence of TDIs in both males and females in different age groups

*Age group*		*Male*				*Female*				*Total*				Chi-square test*			
*years*		*No.*		*%%*		*No.*		*%%*		*No.*		*%%*		χ^2^		*p-value*	
9-10		45		20.4		23		10.4		68		30.8		4.169		0.142	
11-12		36		16.3		23		10.4		59		26.7		1.502		0.220	
13-14		55		25.0		38		17.2		93		42.2		1.729		0.078	
Total		136		61.7		84		38.0		220		99.7		44.188		<0.0001	

*Within age group comparison (male *vs* female)

## OBSERVATION AND RESULTS

From the overall 220 cases of traumatic injuries, in 136 cases (61.8%), the cause of trauma was found to be falls; 45 cases (20.5%) were caused due to collision; 11 cases (5%) were caused due to accident; 7 cases (3.2%) were caused due to violence; 9 cases (4.1%) could not recollect; 12 cases (5.4%) fell under the miscellaneous (Table 1) category. In the age-wise comparison of prevalence of TDIs, the prevalence was more in 13- to 14-year-old males (55 cases; 25%) than females (38 cases; 17.2%) when compared with age groups of 9 to 10 and 11 to 12 years (Table 2). It was observed that the maximum number of TDIs was found with tooth type XI (135 cases; 50%) followed by 21 (71 cases; 25.9%). The most common type of TDI seen was code 2 (152 cases; 55.5%) in both males and females (Table 3); 89.1% TDIs were observed with an overjet of <3.5 mm, 7.7% TDI was observed with an overjet of 3.6-5.5 mm, 2.9% TDI was observed with an overjet of 5.6-8.5 mm, and 0.4% TDI was observed with an overjet of 8.6-11.5 mm in anterior teeth in both males and females. The maximum number of TDIs was found with an overjet of <3.5 mm in both males and females (Table 4). More number of TDIs was found in children with competent lips (206 cases; 93.6%) (Table 5). Prevalence of trauma was found to be more in maxillary arch as compared with mandibular arch in both males and females (Table 6). High prevalence of TDI was seen in children with class I molar relationship as compared with class II and III molar relationship (Table 7).

**Table 3:** **Table 3**: Descriptive statistics showing the comparison of different types of TDIs with type of tooth in males and females

*Trauma* *code*		*11*		*11*		*12*		*12*		*13*		*13*		*21*		*21*		*22*		*22*		*23*		*31*		*31*		*32*		*33*		*33*		*41*		*41*		*42*		*43*	
		*M*		*F*		*M*		*F*		*M*		*F*		*M*		*F*		*M*		*F*		–		*M*		*F*		–		*M*		*F*		*M*		*F*		–		–	
1		9		10		5		*2*		–		–		4		4		1		3		–		–		1		–				–		–		–		–		–	
%		10.6		19.2		17.2		13.3		–		–		9.1		14.8		100.0		100.0		–		–		20.0		–				–		–		–		–		–	
2		46		26		14		6		–		–		30		16		–		–		–		2		4		–		2		–		1		*5*		–		–	
%		54.1		50.0		48.3		40.0		–		–		68.2		59.3		–		–		–		66.7		80.0		–		100.0		–		50.0		100.0		–		–	
3		28		16		9		6		1		–		9		6		–		–		–		1		–		–		–		–		1		–		–		–	
%		32.9		30.8		31.0		40.0		100.0		–		20.5		22.2		–		–		–		33.3		–		–		–		–		50.0		–		–		–	
4		2		–		1		1		–		–		1		1		–		–		–		–		–		–		–		–		–		–		–		–	
%		2.4		–		3.4		6.7		–		–		2.3		3.7		–		–		–		–		–		–		–		–	
5		–		–		–		–		–		–		–		–		–		–		–		–		–		–		–		–	
9		–		–		–		–		–		–		–		–		–		–		–		–		–		–		–		–	
Total		85		52		29		15		1		0		44		27		1		3		0		3		5		0		2		0		2		5		0		0	

## DISCUSSION

This cross-sectional survey has identified a prevalence of 7.3% of TDIs to the permanent anterior teeth among schoolgoing children aged 9 to 14 years in Navi Mumbai (Kharghar-Belapur region). The prevalence noted in the present study was lower as compared with the earlier studies done by Soriano et al^8^ in which the prevalence of TDIs was 23.3%. In a study conducted by Dua and Sharma,^9^ the prevalence of TDIs of 14.5% was found. The prevalence was higher than the study conducted by Nik- Hussein^10^ where a prevalence of 4.1% was found. There may be several reasons for this variation of prevalence between different studies. This could be due to the age of the study subjects, gender, sample size, and criteria used. The male/female ratio was found to be 1.6:1. The male/female ratio was in accordance with the results of a study conducted by Hamdan and Rock^11^ where the male to female ratio was 1.7:1. A possible reason for boys being more prone to TDIs could be their participation and involvement in more aggressive sports and outdoor activities than girls. Violence has also been suggested as a reason of more TDIs in boys. The relatively low prevalence of trauma among girls can also be explained by the fact that girls are in general more mature in their behavior when compared with boys, who tend to be more energetic and inclined toward exuberant outdoor activities. In this study, the peak age to sustain injury was found to be in the age group of 13 to 14 years in both boys and girls, which is similar to the study conducted by Govindarajan et al^12^ in which more number of injuries was found in 10- to 13-year-old children. The cause of injury can differ according to age, gender, level of activity of the child. In our study, the most common cause of traumatic injuries was “fall” followed by collision. The results are in accordance with a study conducted in southern India by Rai and Munshi^13^ and also with the study conducted by Dua and Sharma^9^ where they found “fall” as the most common cause of TDI.

**Table 4:** **Table 4:** Prevalence of TDIs with different overjet measurements in males and females

Overjet		*11*		*11*		*12*		*12*		*13*		*13*		*21*		*21*		*22*		*22*		*23*		*31*		*31*		*32*		*33*		*33*		*41*		*41*		*42*		*43*						*Total*	
–		*M*		*F*		*M*		*F*		*M*		*F*		*M*		*F*		*M*		*F*		–		*M*		*F*		–		*M*		*F*		*M*		*F*		*M*		*F*		*M*		*F*		*M+F*	
<3.5		81		43		28		13		1		–		37		21		1		3		–		5		2		–		2		–		2		5		–		–		157		87		244	
%		95.3		82.7		96.6		86.7		100.0		–		84.1		77.8		100.0		100.0		–		83.3		100.0		–		100.0		–		100.0		100.0		–		–		64.3		35.7		89.1	
3.6-5.5		3		7		1		2		–		–		4		4		–		–		–		–		–		–		–		–		–		–		–		–		8		13		21	
%		3.5		13.5		3.4		13.3		–		–		9.1		14.8		–		–		–		–		–		–		–		–		–		–		–		–		38.1		61.9		7.7	
5.6-8.5		1		2		–		–		–		–		2		2		–		–		–		1		–		–		–		–		–		–		–		–		4		4		8	
%		1.2		3.8		–		–		–		–		4.5		7.4		–		–		–		16.7		–		–		–		–		–		–		–		–		50		50		2.9	
8.6-11.5		–		–		–		–		–		–		1		–		–		–		–		–		–		–		–		–		–		–		–		–		1		–		1	
%		–		–		–		–		–		–		2.3		–		–		–		–		–		–		–		–		–		–		–		–		–		100		–		0.4	
Total		85		52		29		15		1		–		44		27		1		3		–		6		2		–		2		–		2		5		–		–						274	

**Table 5:** **Table 5**: Descriptive statistics showing the comparison of lip competence in the affected children and its association with TDIs

*Lip*		*Male (n = 136)*				*Female (n = 84)*				*Chi-square test*			
*competence*		*No.*		*%*		*No.*		*%*		*χ*^2^		*p-value*	
Competent		128		94.10		78		92.8		0.007		0.930
Incompetent		8		5.90		6		7.2					

In the present study the most common type of TDI was found to be enamel fracture. Garcia-Godoy et al^14^ in their study reported enamel fracture as the most common type of dental injuries, accounting for about 50% of dental injuries. The result derived in our study was unlike the result of the study conducted by Rajab et al^15^ where the most common type of crown injury found to be was enamel and dentin fracture. The relationship between overjet with TDI has been investigated extensively by different authors and has yielded conflicting results. In the present study, no statistically significant relationship has been observed between the overjet and TDI. This result is in accordance with the study by Stokes et al^16^ where no significant difference in the means of overjet sizes between the injury group and the control group was found. Petti et al^17^ reported that individuals with overjet greater than 3 mm were two and half times more at risk when compared with individuals who had a normal overjet. Nevertheless, the present study did not find a positive association, as also asserted in a study by Kumar et al,^18^ who showed an insignificant relation between overjet and TDIs in their study. The reason for such variation in our study can be due to the large population of children with overjet more than 3.5 mm. It is an attention-grabbing question as to why the results of these studies differ so much. It may be because of the investigated populations, the sample sizes, the ways and means of measurement, intraexaminer consistency, reliability, and so on that differ from study to study.

The association between dental trauma and lip coverage is not well defined in the literature. In the present study, no statistically significant relationship has been observed between the lip coverage and TDIs. Some studies have shown this association. In studies conducted by Cortes et al,^19^ more number of injuries was observed with inadequate lip coverage. Traebert et al^20^ did not find any association between inadequate lip coverage and TDI in their study. Glendor reported that these differing results may be due to the interaction between oral predisposing factors and environmental and behavioral factors.^21^ Cases with class I molar relation were found to exhibit large number of TDIs followed by class II div 1 malocclusion. This result is in accordance with the result of the study conducted by Govindarajan et al^12^ in schoolchildren of Tamil Nadu, India. In the study done by Rai and Munshi^13^ in Southern India, the highest number of TDIs was associated with class II div 1. Advanced studies are needed to know the factors that increase the risk of damage to the permanent anterior dentition. Such piece of data is essential to develop and execute effectual preventive approaches for reducing the prevalence of this condition. To understand the complexities of dental trauma epidemiology, additional prospective studies are required on representative populations that will help in implementation of preventive strategies to reduce the frequency of dental trauma.

**Table 6:** **Table 6:** Frequency distribution of trauma between the maxillary and mandibular arches

		*Males (n = 167)*				*Females (n = 107)*				*Total teeth (n = 274)*				Chi-square test*			
*Arch*		*No.*		*%*		*No.*		*%*		*No.*		*%*		χ^2^		*p-value*	
Maxillary arch		160		95.90		97		90.60		257		93.8		9.877		0.002	
Mandibular arch		7		4.10		10		9.40		17		6.2		0.088		0.041	
Chi-square test**		χ^2^		p		χ^2^		p		χ^2^		p					
		101.05		<0.0001		49.158		<0.0001		151.297		<0.0001					

*Within age group comparison; **within-gender comparison

**Table 7:** **Table 7:** Descriptive statistics showing the comparison of molar relationship in the prevalence of TDIs in males and females

*Molar relationship*		*Males*		*%*		*Females*		*%*		*Total (Male + female)*		*%*		*Chi-square*		*p-value*	
Class I		119		87.6		75		89.2		194		88.2		6.243		0.012	
Class II		6		4.4		1		1.1		7		3.1		1.237		0.266	
Class II div 1		10		7.3		8		9.7		18		8.2		0.009		0.923	
Class II div 2		–		–		–		–		–		–		–		–	
Class III		1		0.7		0		–		1		0.5		–		–	
Total		136		100		84		100%		220		100		7.776		0.005	
Chi-square										216.76							
p-value										<0.0001							

## CONCLUSION

Prevalence of TDIs to anterior teeth in schoolchildren of Navi Mumbai (Kharghar-Belapur region) is 7.3%. Males are found to have experienced greater TDIs as compared with females. The most common cause of trauma was found to be fall. The teeth most commonly involved with trauma were maxillary central incisors. The most common type of TDI was enamel fracture. Children (both male and female) in the age group of 13-14 years have experienced highest trauma, when compared with children in the age group of 9-10 and 11-12 years. There was no statistically significant correlation found between overjet, lip competence, and molar relationship with the prevalence of trauma to anterior teeth in the selected population.

This article is significant for pediatric dentists as there are limited studies concerning TDI in young permanent dentition. It offers information about behavioral and anthropometric risk factors for the incidence of TDIs in schoolgoing children. These findings can help pediatric dentists in clinical practice and elaboration of prevention strategies of TDI at the population level.
